# Preliminary Minimum Reporting Requirements for In-Vivo Neural Interface Research: I. Implantable Neural Interfaces

**DOI:** 10.1109/OJEMB.2021.3060919

**Published:** 2021-02-22

**Authors:** Calvin D. Eiber, Jean Delbeke, Jorge Cardoso, Martijn de Neeling, Sam E. John, Chang Won Lee, Jerry Skefos, Argus Sun, Dimiter Prodanov, Zach McKinney

**Affiliations:** University of Melbourne 2281 Melbourne 3010 Australia; Ghent University 26656 Ghent 9000 Belgium; Instituto de Medicina MolecularFaculdade de Medicina, Universidade de Lisboa 70676 Lisbon 1649-028 Portugal; KU Leuven 26657 3000 Leuven Belgium; Hyundai MOBIS 489145 Seoul 16891 South Korea; MetaCell Boston MA 02142 USA; University of California 8783 Los Angeles CA 90095 USA; NeuroElectronics Research Flanders, Imec 71351 3001 Leuven Belgium; BioRobotics Institute and Center for Excellence in Robotics and AIScuola Superiore Sant'Anna 19005 56127 Pisa Italy

**Keywords:** Bioelectronic medicine, neurotechnology, reproducibility, scientific reporting, standardization

## Abstract

The pace of research and development in neuroscience, neurotechnology, and neurorehabilitation is rapidly accelerating, with the number of publications doubling every 4.2 years. Maintaining this progress requires technological standards and scientific reporting guidelines to provide frameworks for communication and interoperability. The present lack of such neurotechnology standards limits the transparency, repro-ducibility, and meta-analysis of this growing body of literature, posing an ongoing barrier to research, clinical, and commercial objectives. Continued neurotechnological innovation requires the development of some minimal standards to promote integration between this broad spectrum of technologies and therapies. To preserve design freedom and accelerate the translation of research into safe and effective technologies with maximal user benefit, such standards must be collaboratively co-developed by the full range of neuroscience and neurotechnology stakeholders. This paper summarizes the preliminary recommendations of IEEE P2794 Standards Working Group, developing a Reporting Standard for *in-vivo* Neural Interface Research (RSNIR).

## I. Introduction

**Fig. 1.**
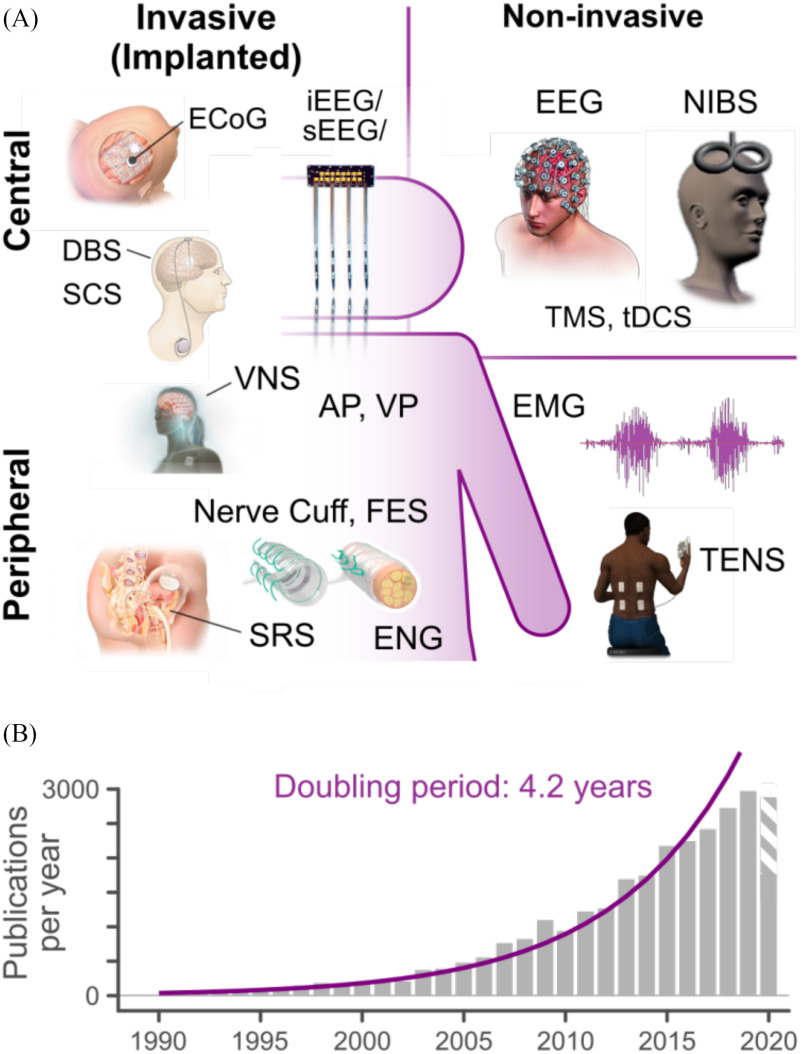
A) Overview of common NI technologies and applications. Neuro-sensing Modalities: EEG (electroencephalography), ECoG (electrocortico-graphy), i/sEEG (intracranial/stereotaxic EEG), EMG (electromyography, ENG (electroneurography). Neuromodulation modalities: AP (auditory pros-thesis), DBS (deep brain stimulation), FES (functional electrical stimulation), NIBS (non-invasive brain stimulation), SCS (spinal cord stimulation), SRS (anterior sacral root stimulation), tDCS (transcranial direct current stimulation), TENS (transcutaneous electrical nerve stimulation), TMS (transcranial magnetic stimulation), VNS (Vagus nerve stimulation), VP (visual prosthesis): B) The accelerating rate of growth for neural interface research (see supplemental methods), in publications per year.

Neural interfaces (NIs) are systems that record and/or modulate the activity of the nervous system (see Fig. 1). A broad spectrum of technological modalities for NIs has been developed over the last 50 years, including both invasive (implanted) and non-invasive systems (Fig. 1A). NIs have been shown to provide therapeutic benefit for a wide range of conditions, as well as providing powerful tools for studying nervous system physiology, improving human-machine interaction, and augmenting human capabilities [1]. The rapid proliferation of neurotechnology in recent years (Fig. 1B) has produced a wealth of devices and systems with advanced neurosensing and neuromodulatory capacities, with a wide range of potential clinical and consumer applications. This diversity of NI technologies, applications, performance metrics, and experimental paradigms – along with the present lack of technological standards and reporting guidelines – has severely limited the transparency, reproducibility, and meta-analysis of this body of literature and hampered its translation into widely adopted and commercially available neurotechnologies.

The effective interpretation, aggregation, and meta-analysis of NI research thus requires more structured and extensive reporting standards to improve the overall ‘information interoperability’ of NI study reports and data. Several related reporting guidelines and initiatives have been enacted in recent years to improve scientific reproducibility and replicability^1^^1^There remain no unified consensus definitions of “reproducibility” and “replicability” – while a majority of engineering and health science disciplines distinguish between *reproducibility* as the ability to derive the same results from the original data/code versus *replicability* as the ability to arrive at the same findings using new data [2], some fields reverse this distinction, with others use the terms interchangeably. This article shall observe the former convention. across the health and cognitive science domains [3]. For example, the Enhancing the Quality and Transparency of Health Research (EQUATOR) network [4] has compiled a list of best-practice reporting guidelines specific to different types of studies, including the CONSORT guidelines for randomized clinical trials [5], the ARRIVE standard for pre-clinical animal trials [6], the PRISMA guidelines for systematic reviews and meta-analyses [7], and more. Collectively, these guidelines articulate the thorough scientific reporting of study protocols, research subjects, and outcomes, but they lack the technological specificity needed to ensure sufficiently detailed descriptions of NI systems, methods, and results for accurate interpretation and replicability.

On a more technical level, regarding the sharing and interoperability of scientific data, the FAIR principles of findability, accessibility, interpretability, and re-usability [8] have been widely recognized in numerous neuroinformatics initiatives, including the International Neuroinformatics Coordinating Facility [9], Neurodata Without Borders[10], the NeuroImaging Data Model [11], ReproNim [12], and the Organization for Human Brain Mapping's Committee on Best Practices in Data Analysis and Sharing (COBIDAS) [13]. However, these guidelines focus primarily on the formatting, analysis, and sharing of *data*, rather than on scientific reporting.

To address this ‘standardization gap,’ IEEE P2794 Standards Working Group (SWG) – spawned from the IEEE Industry Connections Activity on Neurotechnology for Brain-Machine Interfacing [14] — is currently developing a set of Reporting Standards for *in vivo* Neural Interface Research (RSNIR), with the primary objective of improving the scientific quality and transparency of NI research across a wide range of neurotechnological modalities. These Standards aim to establish the technical specificity necessary to achieve thorough interpretability and replicability of NI studies – and thus to improve the positive impact of NI research on the development of safe, effective, and human-beneficial neurotechnologies.

While a primary application of RSNIR will be to improve scientific publications (e.g., via adoption by high-impact journal editors), this standard is intended to serve as a reference for any neurotechnology stakeholder that seeks to improve the rigor and transparency of NI research, including regulatory bodies and funding agencies, as well as for translation of NI research into medical devices. This report previews one such set of guidelines under development, to be included in the forthcoming draft Standard. Constructive feedback is welcomed from all stakeholders, including scientific, commercial, clinical, regulatory, and end-user perspectives.

## II. Scope

The official scope of IEEE P2794 (RSNIR) SWG is to “define the essential characteristics and parameters of in-vivo neural interface research studies (including clinical trials) to be reported in scientific and clinical literature, including both minimum reporting standards and best-practice guidelines.” The RSNIR SWG has defined the scope of NIs to be addressed by the draft Standard to include all engineered systems that directly record bio-signals of neurological origin and/or directly modulate neural activity. “NI research” is defined to include all studies where NI technologies are employed, either as the object of investigation or solely for recording data. More details regarding the scope and organization of SWG P2794 are provided in the Supplementary Materials, Section (§) V.

This article specifically sets forth a preliminary minimum information standard (in the FAIR [8] sense, e.g., [15], [16]) for reporting research involving implanted NIs. The provided recommendations are the most specific regarding electrode-based NIs due to their advanced maturity and broad adoption relative to other NI modalities [17], [18]. Because RSNIR is envisioned to complement existing Standards and consensus guidance documents, the scope of this module does not include aspects of NIs sufficiently specified by existing Standards, such as biocompatibility assessment [19] or characterization of research subjects [5], [6].

## III. Reporting Topics for Implantable Neural Interfaces

As a starting principle, RSNIR-compliant NI study reports should adhere to all applicable reporting guidelines (per the EQUATOR network [4]). The purpose of RSNIR is to expand upon these guidelines by identifying the technological and methodological details necessary to ensure clear, replicable NI reporting. Accordingly, requirements already covered in these ‘parent’ guidelines will not be exhaustively listed here but may be repeated for clarity and context. To promote findable, accessible reporting [8], NI research publications should specify the NI technology(s), neuroanatomic targets, use paradigms /applications, and overall study design in the publicly-accessible metadata (title, abstract, and keywords).

### A. Neural Interfacing Context and Study Aims

To provide sufficient context and rationale, the background/introduction section of NI study reports should clearly identify the fundamental capabilities and limitations in the pertinent technological state-of-the-art and the scientific knowledge gaps addressed by the current study, with reference to authoritative works. Reports should specify the technological or methodological innovation(s) and scientific hypotheses proposed by the study. Testable hypotheses and additional qualitative/descriptive study aims should be stated in relation to the study's primary and secondary outcome measures.

The developmental stage of the study (technology development^1^ vs. animal studies [20] vs. clinical validation [21]) should be identified per Table I. The report should indicate which NI system types and operation modes were investigated, per the IEEE NeuroEthics framework [22]:

•  Recording/sensing
•  Stimulation/neuromodulation
•  Closed-loop control of applications or prosthetic devices
•  Physical/biological modification
•  Neural augmentation and facilitation.

**TABLE I:** Reporting Topics for NI Study Aims and Context

Reporting Topic	High-level Descriptors	Detailed Descriptors
Study Aims and Type	Foundational concept and technology development^I^ (See also [20])	Pre-clinical concept design study (e.g., human cadaver) Modelling & simulation of NI performance Benchtop evaluation of NI capabilities and reliability
	Demonstration in animal models (See also [20])	Acute animal validation and refinement of mechanism Chronic passive safety and reliability Chronic active full system test (ideally in a disease model)
	Human and clinical evaluation (See also [21])	Acute clinical safety & essential performance verification: e.g., partial intra-operative testing Clinical feasibility and pilot studies [28] Clinical validation study (pivotal study / clinical trial) Evaluation & monitoring (post-market)
Intended Application	Neuromodulation (stimulation)	Sensory neuromodulation (e.g., cochlear prosthesis) Motor (Efferent) neuromodulation (e.g., FES)
	Neurosensing (recording)	Diagnostic (e.g., epileptic foci discrimination) Control of an external prosthesis Control of virtual applications
	Closed-loop control or operation	Diagnostic (e.g., H-wave, epilepsy) Targeted delivery of therapy Sensorimotor integration
Physical Modality / Technology	Electrical	quasi-electrostatic (µs-s timescales), tDCS electrodynamic (fs-ns timescales, e.g., [42]), single- or multi-unit recording, field potential recording, ECoG, EEG.
	Magnetic and Electromagnetic	fMRI, TMS, Magnetoencephalography (MEG)
	Optical and Infrared	Optogenetic stimulation Voltage-sensitive or calcium-sensitive recording fNIRs, IR stimulation
	Acoustic	Focused ultrasound stimulation
Target Neural Structure(s)/Pathway(s)	Central Nervous System (CNS)	Targeted brain or spinal cord region(s) to be named per [34], [35], [43]
	Peripheral Nervous System (PNS)	Targeted division(s)^II^ and neuroanatomical structures to be named per [34], [35], [43]
	Enteric Nervous System (ENS)	Targeted neural structures to be named per [34], [35], [43]

^1^Lab bench and in vitro studies are beyond the official *in vivo* scope of RSNIR. Recommendations given here as reference, for complementarity to *in vivo* studies.

^2^The PNS is classically divided into somatic and autonomic divisions, with the autonomic further delineated into parasympathetic and sympathetic sub-divisions.

These loosely align with the brain-computer interface application scenarios originally proposed by Wolpaw and colleagues [23] and expanded upon in [1]: replacing, restoring, enhancing, supplementing, improving, and studying neurological function. Finally, the NI description should specify the target neuroanatomical structure(s) and device-tissue interface type/region.

### B. NI Experimental Design and Outcome Measures

As a guiding principle, all aspects of experimental designs featuring NIs should be described in sufficient detail to permit replication by other researchers, provided use of the same NI system(s) and experimental setup. All NI studies must comply with consensus standards of ethical conduct, including local regulations, institutional review board approval, and the Declaration of Helsinki [24]. Tables I and II list essential study characteristics to be reported, as outlined below.

**TABLE II:** Reporting Topics for NI Experimental Design and Outcome Measures

Reporting Topic	High-level Descriptors	Detailed Descriptors
Animal Models	Fundamental characteristics	Number and type of subjects involved, including justification of sample size (both projection and actual numbers). Species/ strain and genetic background, bodyweight, administered genetic manipulations (if relevant).
	Husbandry and housing conditions	light/ dark schedule, environmental enrichment, experimental location. All administered drugs and drug doses, including administration routes^I^.
	Training and behavior (if relevant)	Training, reward, and performance assessment methods.
Human Subjects	Eligibility and recruitment	Complete list of inclusion and exclusion criteria, Criteria used to allocate subjects to experimental groups Recruitment methods for subjects and controls
	Demographic characteristics	Number and type of subjects involved, Subject age and gender Statistical justification of sample size, including “convenience sampling”
	Relevant clinical history	Timelines of disease onset and symptom presentation All administered drugs and drug doses, including administration routes. Any other parallel treatments^I^.
Interventions	Description of all interventions applied	(procedures, devices, treatment programs, surgical procedures, etc.)
	Sequential timeline of interventions	including sequences and interrelations, Randomization for cross-over / within-subjects-type designs
	Location and setting of the experiments	(e.g., clinic, home setting, animal laboratory or home cage)
	Experimental Equipment (Excluding NI)	Any specialized medical equipment used during the experiments, Experimental stimulator / actuator and system information, Any other sensors or actuators used to assess the performance of the NI: Vendor, make and model, control or acquisition system software and version
Stimulus Description	Visual Stimuli^II^	Background illumination level (e.g., scotopic or in units of cd / m2), Adaptation state of the experimental subject (e.g., dark-adapted), Duration of the stimulus including any adaptation or masking procedures, Approximate retinotopic location of the stimulus presentation (e.g., foveal) Frame-rate and luminance range of the display.
	Auditory Stimuli^II^	Background and stimulus sound levels, Stimulus presentation (e.g., monaural, binaural), Tone frequency and duration.
	Tactile Stimuli	Similarly, for tactile stimuli, the stimulus type (e.g., vibratory, single-pulse, von Frey, etc.), intensity (in mm/s) and other properties should be reported.
	Other Stimuli	For more complex stimuli, such as movies or sequences of spoken words, examples should be provided as supplementary data.
Outcome Measures	Basic signal quality metrics for NIs	(e.g., signal-to-noise ratio)
	Usability and patient satisfaction scores.	For animals research, these may include behavioral assessments e.g., [44]
	Computation of derived measures	References to established measures and formulas for novel measures
Statistics	Identification of dataset(s) between which each comparison was conducted	Description and rationale for data grouping provided (e.g., between vs. within-subjects comparisons).
	Derivation for each datum	Time point(s) for data sampling Single measurement or aggregated measures.
	Other statistical methods	Methods used to examine subgroups, Assessment of multi-variate interactions, Control for confounding and missing data, Mitigation of potential sources of bias.

^1^This is important, as many drugs have effects on the nervous system which may influence NI performance, e.g., [45].

^2^For more complex stimuli, such as movies or sequences of spoken words, examples should be provided as supplementary data.

#### 1) High-Level Study Design

The NI study description should first identify the overall experimental paradigm(s) using established study design taxonomy terminology such as prospective/retrospective cohort study, single/double-cohort, randomized controlled trial (RCT), or case-control study [25], [26]. Within-subjects designs (where each participant serves as their own control, such as n-of-1 case studies [27]) are common for early clinical and pre-clinical NI research, with the main motivation to demonstrate proof-of-concept and/or subject-specific safety and efficacy of the NI prior to conducting large-scale clinical trials. Given the high tendency for individual variability, this approach demands a detailed description of the clinical and demographic characteristics of all subjects (Table II). Follow-up data collection to monitor the clinical evolution after experimental intervention is highly encouraged.

Subsequent pilot [28] and larger-scale clinical studies evaluating an intervention's effectiveness with respect to established standard therapy(s) for broader user populations typically employ between-subjects study designs, such as the “gold standard” RCT. Important for these types of experiments is the definition and recruitment of a representative control group. Blinded assessment of outcomes is strongly encouraged*.* In “crossover” designs featuring multiple interventions administered in serial, randomization of intervention sequence between subjects is advised, with a sufficiently long “washout” period to combat carryover effects (such as improved performance due to longer exposure to the NI). Such experimental designs can also be used in animal studies.

For all study designs, subjects should be characterized in detail per Table II, including all inclusion/exclusion criteria, recruitment methods, and group allocation. Baseline outcome measures should be noted before the start of intervention, along with other relevant clinical and demographic characteristics.

#### 2) Description of Intervention(s)

All interventions, including procedures, NI devices, treatment programs, and surgical procedures, must be described in detail to ensure reproducibility. Stimulation and recording protocols, including the conditions under which the experiment was conducted, must be reported. If visual, auditory, tactile, or other sensory stimuli were used in either experimental or control conditions, these stimuli must be described per Table II. Whenever the experimental design involves behavioral assessments, potential behavioral biases and mitigation strategies (whenever applicable) should be reported (e.g., human handedness, education, expectations about the study).

#### 3) Outcome Measures and Statistical Analysis

All outcome and performance assessment measures – both NI-derived and otherwise – must be precisely defined. The selection and relevance of all such measures to the study aims and hypotheses should be justified. Basic signal quality metrics for NI data (e.g, signal to noise ratio) are recommended, as are usability and user satisfaction scores.

All statistical analyses should be reported according to pertinent reporting guidelines and best practices (e.g., [4], [29], [30]). Reporting of data-processing and statistical methods must be sufficient to reproduce the presented results from raw data. The data set(s) between which each statistical comparison was conducted (e.g., between vs. within-subjects) must be clearly reported and justified. Where feasible, intended analyses of outcome measures should be documented and disclosed prior to data collection in order to maximize transparency and the statistical validity of the results obtained and minimize the opportunity for so-called ‘p-hacking’ [3].

### C. Description of the Neural Interface

Insufficiently detailed reporting of NI device/system characteristics is the biggest barrier to the interpretability, replicability, and meta-analyzability of NI research – especially clinical studies. To overcome this barrier, researchers must provide a thorough description of the NI (per Table III), including specification of the applied stimuli and/or recording procedures. These parameters are critical to comparing NI performance across technologies, devices, and cohorts.

**TABLE III:** Reporting Topics for NI Physical Device Properties

Reporting Topic	High-level Descriptors	Detailed Descriptors
Intended Device Service Life	Acute	Duration of intended use: ≤ 24 hours (e.g., intra- and peri-operative use)
	Short-Term (Sub-acute and Sub-Chronic^I^)	Duration of intended use: 24 hours to 28 days (including acute testing of devices intended for short-term implantation).
	Chronic	Duration of intended use: > 28 days (including acute tests of devices intended for chronic implantation)
Level of Invasiveness	Implanted	Minimally-invasive^II^, including endovascular (e.g., [47],[48]) vs. Extracellular (e.g., LFP, DBS) vs. Intracellular
	External (non-implanted)	Transcutaneous vs. Percutaneous or Semi-invasive (e.g., [49])
Implantation / Positioning Procedure	Anatomical positioning	Recording tip coordinates in stereotaxic coordinates or with reference to anatomical landmarks (gyri/sulci, lambda/bregma, branching points or major blood vessels for peripheral nerves). See Supplement, §VI.E.
	Fixation and adjustment procedures	Intraoperative and/or postoperative, including anchoring site and fixation.
	Locations of secondary connections	e.g., distant return, patient reference potential^III^
	Lead-wire / connector positioning and fixation	Include battery / antenna / percutaneous plug placement as needed.
Electrode / Transducer Design (see [50])	Commercially available device specifications^IV^	Vendor/model information, including firmware and graphical software versions System configuration, operational parameters (e.g., stimulation settings), and device modification(s)
	Type, number, and arrangement of electrodes/transducers	Transducer type: e.g., microwire, micromachined, or polymer-based (see [50]) Overall array design (see [51], Supplement §VI.D) Spacing between electrodes / recording shanks.
	Geometry of individual electrodes/transducers	Recording site footprint (e.g., diameter, width x length). See Supplement §VI.G.
	Lead / connector geometry	Shank / guide cannula dimensions (length, diameter, cross-section) Connector type. See Supplement §VI.G.
Device Materials and Fabrication (see [50])	Electrode / transducer materials	Core conductive material (See Supplement, §VI.F). Plating materials or surface treatments (if relevant) Report the materials used for secondary connections as well.
	Other materials	Lead / connector materials, NI device encapsulation materials^V^, Materials for fixation screws, sutures, or other support materials
	Mechanical properties^V^	Stiffness of the transducer / electrode array carrier. Stiffness of any connectors / lead-wires.
	Fabrication methods^V^	Microfabrication techniques & parameters (e.g., electrodeposition methods) see [50]
	Sterilization protocol	Sterilization mechanism (e.g., autoclave, ethylene oxide, gamma irradiation, plasma) and process parameters, with reference to Standard protocols (see [52])
Electrical Properties (see [50])	Electrode impedance	Impedance measurement method (see Supplement §VI.H) – Measured at 1kHz and intended NI operating frequencies [53], [54]
	Stimulus Driver properties	Dynamic range, frequency response and equivalent parallel (or series) resistance and reactance.
	MRI compatibility	As relevant to intended application(s), compatibility w MRI [55] and/or other imaging modalities should be reported, w/ reference to pertinent testing standards (e.g., [56]).
	Power requirements	For implanted NIs, detail minimum required flows of power and data (bitrates) for NI system function, Estimate of implanted system lifespan, Considerations of tissue heating

^1^ISO 10993 [19] loosely defines the terms “sub-acute” (> 24hr, <14d) and “sub-chronic” (14-28d) in the context of systemic toxicity evaluation.

^2^Here, we use “minimally-invasive” to describe implanted NIs for which tissue or organ barriers such as the meninges or perineurium are not breached.

^3^See main text footnote 2 regarding the use of the term ‘reference’ vs ‘ground’, and also Supplementary Materials, Section VI.C.

^4^Reporting of other details can be referenced to literature, provided those details have been measured in an equivalent (intraoperative) environment.

^5^The mechanical and electrochemical properties of NIs are critical to their long-term safety & efficacy and influenced by fabrication techniques. See [50], [57], [58].

**Fig. 2.**
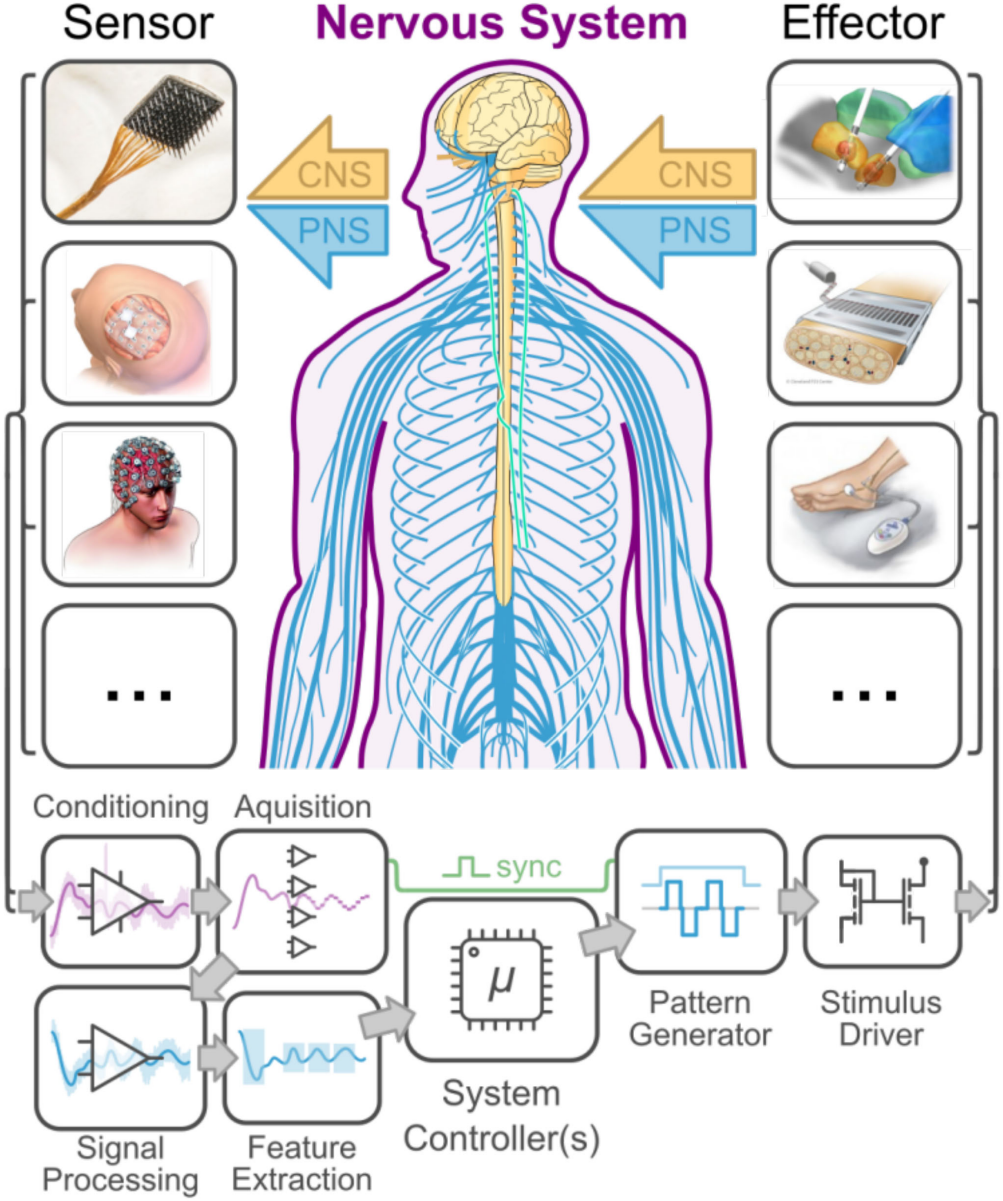
Block diagram of a prototypical NI system architecture. Sensors and effectors may interface invasively or non-invasively with the central or peripheral nervous system (CNS / PNS). Neural sensing components will almost always include hardware signal conditioning, digital-to-analog conversion, digital signal processing, and feature extraction. Neuromodulation components include waveform selection and generation and the output drive to the stimulus end effector. Sensors, from top to bottom: high-density intracortical (Utah) array, ECoG array, EEG. Effectors: deep brain stimulation, peripheral nerve array (FINE, [46]), and transcutaneous stimulation.

Fig. 2 shows a block diagram of a generic closed-loop NI system architecture which includes transducers (electrodes), signal acquisition and processing for neural recording, and stimulus generation and delivery for neuromodulation. The characteristics of all of these modules are essential for interpreting NI performance; essential reporting parameters for NI transducers and hardware are given in Table III, and essential reporting parameters for NI signal acquisition and processing is given in table IV. Diagrams such as Fig. 2 are essential for communicating the overall plan for a given NI approach and application, and we encourage their use for describing both the NI under test and the experimental context in which the NI is deployed. For custom experimental devices (including modified devices), authors should also provide a labelled diagram showing electrode / transducer sizes and locations.^2^^2^Electrode names like ‘anode’ and ‘cathode’ lead to confusion in the context of biphasic charge-balanced electrical stimulation to and should be avoided. Similarly, the labels ‘active’ or ‘reference’ imply assumptions about where activity or activation is occurring which may not be satisfied. For current-controlled stimuli, the term ‘return’ is clearer and should be preferred. For recording, ‘reference’ is to be preferred to other terms as this is the potential connected to a galvanically isolated recording device. As patients must never be connected to earth, by any means, terms such as ‘earth’, ‘neutral’, ‘safety ground’, or ‘building ground’ must not be used. We suggest using electrode / transducer labels like E1, E2, E3, etc.

**TABLE IV:** Reporting Topics for NI Signal Processing Properties

Reporting Topic	High-level Descriptors	Detailed Descriptors
Target Physiological Signal	High-resolution recording (e.g., single-unit)	Stability over time Cell type specificity and bias in recording
	Population-averaged recording (e.g., LFP, EEG, ECoG, ENG)	Stability over time, Spatial and temporal resolution of the observed signal Cellular origin of the observed signal (e.g., [59])
	Neuromodulation	Spatial resolution of the imposed signal Cell type specificity of the evoked response Safety limits (power and/or current density [58]) and dynamic range Observed threshold ranges For nerve block: what is the purported mechanism of blockade?
Hardware Conditioning and Acquisition	Filtering	Input gain, anti-aliasing filter time-constant^I^ Hardware artifact rejection and saturation range^II^
	Analog to digital conversion	Sampling rate, dynamic range, resolution (in bits or µV)
	Output channels	Number of channels Map from sensor signals to channels (electrode/transducer identifiers) Output channel characteristics including estimates of cross-talk
Signal Processing	Filtering	Filter type (high-pass, low-pass, band-pass, notch), implementation (e.g., passive filter, active Butterworth, Bessel, etc.), filter order, and rationale^III^. Details of any applied averaging or normalization Details of any nonlinear filtering e.g., Kalman filtering. Visualize input and output signal characteristics
	Artifact removal	Algorithm and parameter estimation Order of applied signal-processing steps^IV^
Feature Extraction	Frequency-domain transformation	Define all analysis bands used Define wavelets & analysis windows for time-resolved spectral power estimation (e.g., [60])
	Spatial transformation	Mathematical transformation(s) used (e.g., inverse source localization)
	Coordinate transformation	Mathematical transformation(s) used (e.g., PCA/ICA, SVD [61]). Input and output signal characteristics
	Datatype conversion	(e.g., spike detection and sorting, autoregressive model fitting) Input and output signal characteristics Sensitivity to input noise, signal-to-noise ratio.
Classification and Decision-making	Classification analyses	Classifier architecture (e.g., SVM, K-means, CNN, etc.: see [62]). Model parameters & hyperparameters Initialization, Convergence criteria
	Inference of mental states	The states identified must be clearly specified. The usual ‘expert’ rules applied for offline analysis should be referenced (e.g., [63])
	Control Algorithms for effectors / stimulus delivery	Including algorithms for closed-loop control, “model-in-loop” control.
Stimulus Waveform Generation	Timing of stimulus delivery	Patient / subject control, Trigger signals or events Synchronization with recording equipment
	Stimulus type	(e.g., current, voltage, optical, acoustic, etc.). For electrical stimuli, equivalent standard rectangular current-controlled stimulus.
	Timing of individual stimuli	Phase width, pulse shape, leading phase, inter-phase gaps. Stimulus frequency, number of discrete stimuli per stimulation period Shorting / charge-balancing procedures for electrical stimuli.
	Output channels	Number of independently addressable stimulation channels available, Electrode/transducer configurations used.

^1^For recording physiological signals, transient artifacts can mimic physiological signals when filtered through high-pass filters higher than first-order. To avoid this, characterizing any such filter by a single time constant value can ensure this good practice has been enforced.

^2^If recording signals reach saturation during regular use (e.g., due to stimulation artifacts), this should be noted along with the expected duration of invalid signal.

^3^Adequate filter specification is necessary to extract useful signals from noisy neurodata but is frequently underreported. Quite often, filters are used with non-linear group velocity transfer functions, and analysis methods are applied afterwards which assume linear group frequency transfer functions. We have also seen cases where non-causal filtering (e.g., MATLAB's filtfilt) is applied in such a way as to cause responses to precede stimulus, which is obviously nonsensical.

^4^In principle, the order of linear signal processing steps is not important. In practice, malfunction, artefacts, and other sources of confusion are more easily identified in the frame of an orderly description. See Supplemental Materials, Section VII.

The placement and positioning of the NI are critical to NI effectiveness (see [31]–[33]) and must be carefully reported (including the transducer, connectors, and any implanted electronics). Anatomical structures should be specified with reference to a widely-accepted formal vocabulary such as the Federative International Program for Anatomical Terminology [34] or recognized anatomical atlases (e.g., [35], [36]). Implantation and device positioning procedures must be described, including the location of each component relative to anatomical landmarks, expected error margins, and any criterion for surgical re-positioning or exclusion. Describe any procedures carried-out to confirm device position during or after concluding the experiment (e.g., histology, CT imaging). Finally, for research concerning entire implanted NI systems (as opposed to investigations of NI components), expected and observed implant lifespans should be reported, as well as any observed or predicted failure modes (e.g., [37]).

From a clinician, end-user, or regulatory perspective, the algorithms used for signal-processing, stimulus generation and closed-loop control are as much a part of a NI as the underlying hardware. Reporting of these aspects of NI systems must be conducted to the same level of rigor as reporting of the physical interface; essential reporting parameters are given in Table IV. For neuro-sensing NIs, an unambiguous description of how signals from the electrodes / transducers are processed into recording channels is necessary. For novel NIs using recording approaches which might not be familiar to the wider NI community, the biophysical basis for the observed signals and measurement approach should be justified. Similarly, for novel neuromodulation NI approaches, the mechanism of the modulation of nerve activity should be described.

Algorithms used for signal conditioning, pre-processing, and analysis must be clearly reported and referenced. Providing public repositories containing implementations with representative data sets is recommended. Inputs and outputs should be clearly specified, including confidence interval estimates (e.g., via bootstrap analysis of noisy input data, [38]). Existing standards for signal-processing research (e.g., [39]) should be applied.

### D. Neural Interface Results and Discussion

NI research reports should clearly and succinctly present the results of all analyses described in the methods (including primary and secondary outcomes), plus any additional post-hoc analyses (identified as such), in a manner that accurately summarizes and represents the full data set(s) analyzed, according to established biostatistical best practices [29], [30]. Graphical data representation (figures and tables) is preferred to text. Numerical values displayed in figures should be either incorporated in the figure, given in a corresponding table, or in the supplemental materials. Wherever applicable (including aggregated measures and descriptive statistics), measurement variability and uncertainty should be quantified with standard measures (standard deviations, confidence intervals, etc.). Likewise, all comparisons conducting using inferential statistics should report statistical significance (or non-significance) and effect size. Where parametric statistics are used, the normality of data distribution should be reported. Rationale should be provided for the exclusion from presented analysis of any data collected within the same protocol. Measures of NI signal quality (e.g., signal-to-noise ratio) or essential performance are *strongly* recommended, along with presentation of example raw data.

All unexpected or adverse events (e.g., device failures or explantations, subject withdrawal, unplanned animal deaths, etc.) should be reported. Observed technical issues and complications should also be reported, including all mechanical, electrical, or software failures (broken electrodes, connections, etc.).

Discussion of results should address the following topics:

• To what extent do the results confirm the study hypothesis/es, and how do they fulfill the study objectives?
• The distinction between statistical and clinical/functional significance, with reference to the observed effect size, uncertainty, and minimal clinically important difference.
• The fundamental novelty and/or significance of the findings with respect to the current state of the art, scientific body of knowledge, and/or field of potential applications. Comparisons to results of previous similar studies are encouraged, with attribution of notable similarities differences.
• The applicability and generalizability results to the intended NI users and applications, addressing concepts of validity (internal vs. external; construct; content; face)
• Discarded data collected according to the study protocol but excluded the final presented results/analysis.
• Identification of key study limitations pertaining to the subject population, animal model, and/or experimental paradigm:
  ○ Uncontrolled and potentially confounding factors
  ○ Precision and uncertainty of measurements, including intra-and inter-subject variability
  ○ The stability of neural recordings and/or stimulation parameters over the time course of the study
  ○ Potential sources of biases in the subject recruitment /enrollment process.
  ○ Study withdrawal rates
• Limitations of the presented technology/approach with respect to present or future application(s).
• Key challenges to the future development and application of the presented technologies, including usability considerations and open questions for further investigation.

## IV. Discussion

As a preview of the proposed IEEE P2794 (RSNIR) draft Standard, this document has outlined minimum reporting requirements to ensure adequate transparency, reproducibility, and replicability of *in vivo* research involving implantable NIs, in line with recommendations from the National Academies of Sciences [64]. In this way, RSNIR aims to complement existing scientific and clinical reporting guidelines by adding a layer of specificity to implantable NI technology. Most of these recommendations apply to all NI technology (including non-invasive modalities), and the RSNIR SWG is currently working to adapt these requirements to such technologies, including EEG-based BCIs.

In addition to high-level scientific reporting guidelines (EQUATOR etc.), RSNIR will be supported by a network of complementary NI-relevant Standards under current development, including IEEE P2731 (Unified Technology for Brain-Computer Interfaces) and P2792 (Therapeutic Electrical Stimulation Waveforms). For medical NI technologies, RSNIR also aims to facilitate compliance with foundational medical device standards such as ISO 14971 (risk management), ISO 13485 (quality management systems), and IEC 60601 (safety and essential performance requirements), as well as neurotechnology-specific standards such as ISO 14708 and EN 45502 (active implantable medical devices for surgery).

The future impact of RSNIR in promoting high-quality neuroscience and neurotechnology development will depend critically on its widespread adoption by a variety of institutions that define incentives across academic, commercial, and clinical domains, including high-impact scientific publications, funding agencies, regulatory bodies (including clinical trial registries [40]), and/or medical payers. To promote such adoption, the draft Standard will seek to support an ‘ecosystem of information interoperability’ that serves the needs and objectives of all neurotechnology stakeholders, including aforementioned institutions as well as researchers, developers, clinicians, and end users.

To facilitate adoption at different levels of technological maturity (e.g., Technology Readiness Level [41]), the draft RSNIR will apply the principle of *indirect reporting,* whereby reporting requirements may be fulfilled via reference to previous publications or documents, provided that all required details are contained in the primary publication (including supplemental materials) and all others *directly* cited therein.

Regarding potential adoption by commercial entities, the draft RSNIR will seek to accommodate the proprietary nature of some NI system design details, by allowing the study replicability criterion to be fulfilled on a system-dependent basis, requiring the use of commercial hardware or software. In such cases, public assurance of the NI system's basic safety and performance may be achieved via third-party certification according to official testing Standards (UL, ASTM, CE-marking, etc.). To make the official RSNIR usable and useful at all stages of research & development (technological maturity), feedback to this article and participation in the RSNIR SWG are welcomed from all such stakeholders.

## Supplementary Materials

Supplementary materials
